# Update Expertenzertifikate: Ergänzende Strukturmerkmale und Modifikation *„Kopf-Hals-Onkochirurgie“* sowie Neuinitiative *„Nasennebenhöhlen- und Schädelbasis-Chirurgie“*

**DOI:** 10.1007/s00106-025-01707-z

**Published:** 2026-01-23

**Authors:** Thomas K. Hoffmann, Thomas Deitmer, Martin Jäckel, Andreas Neumann, Burkhard M. Lippert, Timo Stöver, Jens Peter Klußmann, Christian S. Betz

**Affiliations:** 1 Klinik für Hals‑, Nasen‑, Ohrenheilkunde, Kopf- und Halschirurgie, Universitätsklinikum Ulm, Frauensteige 12, 89070 Ulm, Deutschland; 2 Deutsche Gesellschaft für Hals-Nasen-Ohren-Heilkunde, Kopf- und Hals-Chirurgie e. V., Friedrich-Wilhelm-Str. 2, 53113 Bonn, Deutschland; 3 Klinik für Hals‑, Nasen‑, Ohrenheilkunde, Helios Kliniken Schwerin, Wismarsche Straße 393–397, 19055 Schwerin, Deutschland; 4 Klinik für Hals‑, Nasen‑, Ohrenheilkunde, Lukas Krankenhaus, Preußenstraße 84, 41464 Neuss, Deutschland; 5 Klinik für Hals‑, Nasen‑, Ohrenheilkunde, SLK-Kliniken Heilbronn GmbH, Am Gesundbrunnen 20, 74078 Heilbronn, Deutschland; 6 Klinik für Hals‑, Nasen‑, Ohrenheilkunde, Universitätsmedizin Frankfurt a.M., Goethe-Universität, Theodor-Stern-Kai 7, 60590 Frankfurt a.M, Deutschland; 7 Klinik für Hals‑, Nasen‑, Ohrenheilkunde, Kopf- und Halschirurgie, Universitätsklinikum Köln, Medizinische Fakultät der Universität zu Köln, Kerpener Str. 62, 50937 Köln, Deutschland; 8 Klinik für Hals‑, Nasen‑, Ohrenheilkunde, Kopf- und Halschirurgie, Universitätsklinikum Hamburg-Eppendorf, Martinistr. 52, 20246 Hamburg, Deutschland

**Keywords:** Kopf-Hals-Chirurgie, Kopf-Hals-Onkochirurgie, Nasennebenhöhlenchirurgie, Schädelbasischirurgie, Epertenzertifikat, Head and neck surgery, Head and neck oncosurgery, Paranasal sinus surgery, Skull base surgery, Expert certification

## Abstract

Die Deutsche Gesellschaft für Hals-Nasen-Ohren-Heilkunde, Kopf- und Hals-Chirurgie e. V. (DGHNO-KHC) und die Deutsche HNO-Akademie (DAHNO) haben unter Mitwirkung der entsprechenden Arbeitsgemeinschaften *Expertenzertifikate* für den Teilbereich „Kopf-Hals-Onkochirurgie“ und nun auch den der „Nasennebenhöhlen- und Schädelbasis-Chirurgie“ entwickelt. Ziel ist es, in Analogie zu internationalen Standards, die Expertise der Antragstellenden für den jeweiligen Teilbereich darzustellen. Für die „Kopf-Hals-Onkochirurgie“ wurde das Qualifikationsmerkmal „Tätigkeit in einem von der Deutschen Krebsgesellschaft/DKG zertifizierten Kopf-Hals-Tumorzentrum“ um das Kriterium „oder vergleichbarer Einrichtung (Strukturmerkmale: regelmäßige interdisziplinäre Fallkonferenz und Zusammenarbeit mit Hauptkooperationspartnern mit Vorhaltung definierter Behandlungspfade)“ ergänzt und hinsichtlich Fortbildungs- bzw. Studienteilnahme modifiziert. Für das Expertenzertifikat „Nasennebenhöhlen- und (anteriore) Schädelbasis-Chirurgie“ wurden gemeinsam mit der Arbeitsgemeinschaft Rhinologie/Rhinochirurgie (ARHIN) und der Arbeitsgemeinschaft Schädelbasis- und kraniofasziale Chirurgie (ASKRA) die Kriterien für ein entsprechendes Logbuch entwickelt. Die Zertifikate können zum Nachweis der individuellen Expertise genutzt werden. Die praktische Umsetzung erfolgt durch eine unabhängige Zertifizierungsstelle (ClarCert GmbH) im Auftrag der DGHNO-KHC und beurteilender Mitarbeit der DAHNO sowie der jeweiligen Arbeitsgemeinschaften. Anträge können durch Mitglieder der DGHNO-KHC oder der DAHNO ab sofort für die genannten Expertenzertifikate gestellt werden. Weitere Zertifikate befinden sich in Vorbereitung.

## Abstract (en)

The German Society of Otorhinolaryngology, Head and Neck Surgery (DGHNO-KHC) and the German ORL Academy (DAHNO), in cooperation with the relevant working groups, have developed expert certificates for the field of “Head and Neck Surgical Oncology” and now also for “Paranasal Sinus and Skull Base Surgery”. The aim is to document the applicants’ expertise in the respective subfield, in line with international standards. For the “Head and Neck Surgical Oncology” certification, the qualification criterion “activity in a head and neck tumor center certified by the German Cancer Society (DKG)” was supplemented by the criterion “or comparable institution (structural features: regular interdisciplinary case conference and collaboration with main cooperation partners with defined treatment pathways)” and modified with regard to further training or participation in studies. For the expert certificate “Paranasal Sinus and (Anterior) Skull Base Surgery,” criteria for a corresponding logbook were developed in collaboration with the Working Group for Rhinology/Rhinosurgery (ARHIN) and the Working Group for Skull Base and Craniofacial Surgery (ASKRA). These certificates can be used to demonstrate individual expertise. Practical implementation is carried out by an independent certification institute (ClarCert GmbH) on behalf of the DGHNO-KHC and in cooperation with DAHNO and the respective working groups. Applications for the aforementioned expert certificates can now be submitted by members of the DGHNO-KHC or the DAHNO. Further expert certificates are in preparation.

Hochspezialisierte chirurgische Behandlungen bspw. in der Kopf-Hals-Onkologie, Otologie oder Rhinologie werden durch technische Innovationen, Weiterentwicklung operativer Techniken sowie Vor‑, Begleit- und Nachbehandlungen immer komplexer. Sie erfordern eine langjährige Ausbildung, die aufgrund ihres Umfangs häufig erst nach der Facharztweiterbildung angeschlossen wird. Durch den Wegfall der Zusatzweiterbildung „Spezielle Kopf-Hals-Chirurgie“ seit 2003 (mit Übergangsfristen bis 2014) gibt es derzeit – mit Ausnahme der Zusatzbezeichnung „Plastische und Ästhetische Operationen“ – keine Möglichkeit, eine besondere chirurgische Expertise über formelle Qualifikationen erkennbar zu machen. Gleichzeitig werden aber bspw. bei der Zertifizierung von Zentren spezifische chirurgische Kompetenzen gefordert und konkret abgefragt. Es besteht damit ein Bedarf für den Nachweis chirurgischer Spezialisierungen und deren transparenter Überprüfung. Die hiernach qualifizierten Personen sollen entsprechende Zertifikate erhalten. Die Deutsche Gesellschaft für Hals-Nasen-Ohren-Heilkunde, Kopf- und Hals-Chirurgie e. V. (DGHNO-KHC) und die Deutsche HNO-Akademie (DAHNO) haben unter Mitwirkung der Arbeitsgemeinschaft Onkologie die Kriterien für den Erwerb des Expertenzertifikats „Kopf-Hals-Onkochirurgie“ modifiziert und gemeinsam mit der Arbeitsgemeinschaft Rhinologie/Rhinochirurgie (ARHIN) und der Arbeitsgemeinschaft Schädelbasis- und kraniofasziale Chirurgie (ASKRA) ein Expertenzertifikat für den Teilbereich der „Nasennebenhöhlen- und (anteriore) Schädelbasis-Chirurgie“ entwickelt.

## Kriterien

Voraussetzung für den Erwerb der Zertifikate ist die Erfüllung der nachfolgend genannten Inhalte:

### „Kopf-Hals-Onkochirurgie“

**Abb. 1:**
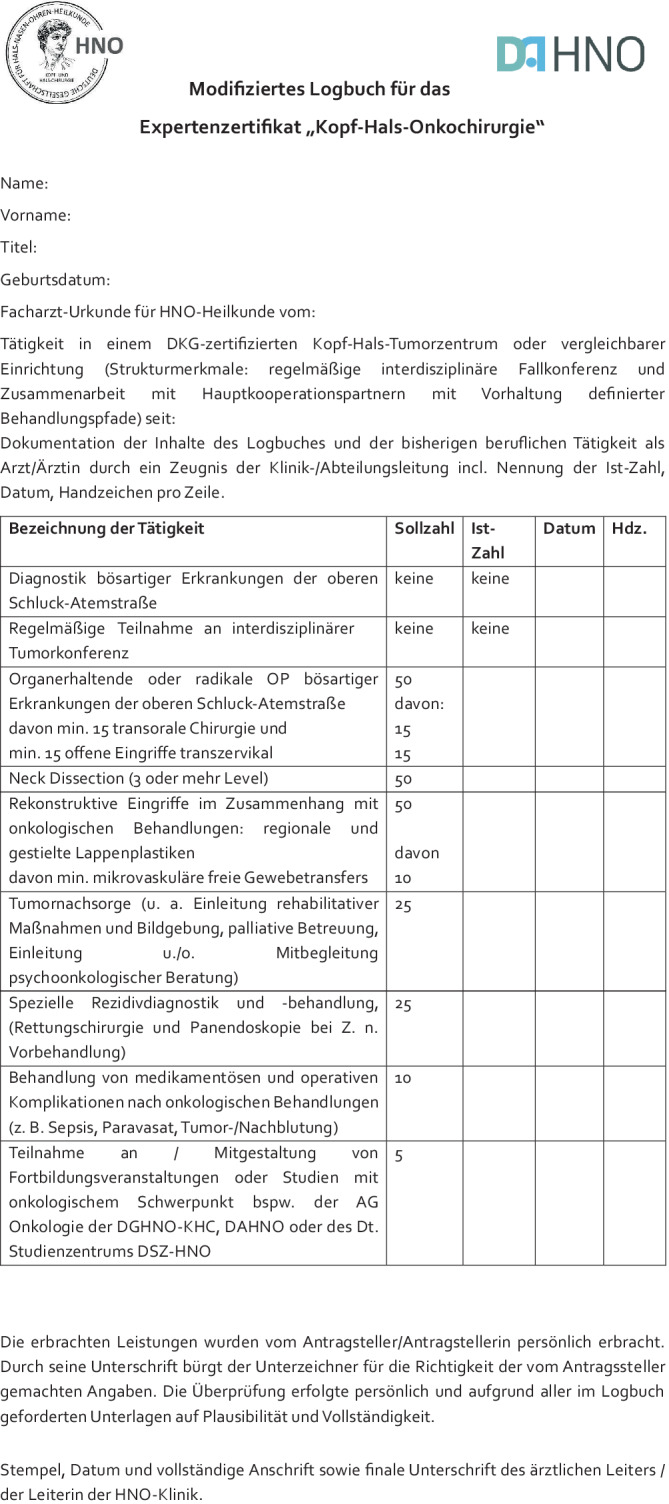
Modifiziertes Logbuch mit Kriterien zum Erwerb des Expertenzertifikats „Kopf-Hals-Onkochirurgie“

In Bezug auf die bereits festgelegten und publizierten Kriterien [1] wurde für das Qualifikationsmerkmal „Tätigkeit in einem von der Deutschen Krebsgesellschaft/DKG zertifizierten Kopf-Hals-Tumorzentrum“ um das Kriterium „oder vergleichbarer Einrichtung (Strukturmerkmale: regelmäßige interdisziplinäre Fallkonferenz und Zusammenarbeit mit Hauptkooperationspartnern mit Vorhaltung definierter Behandlungspfade)“ ergänzt. Zudem ergab sich eine Modifikation hinsichtlich der Fortbildungs- bzw. Studienteilnahme, wie folgt: „Teilnahme an/Mitgestaltung von Fortbildungsveranstaltungen oder Studien mit onkologischem Schwerpunkt bspw. der AG Onkologie der DGHNO-KHC, DAHNO oder des Dt. Studienzentrums DSZ-HNO“ (Abb. 1).

**Abb. 2:**
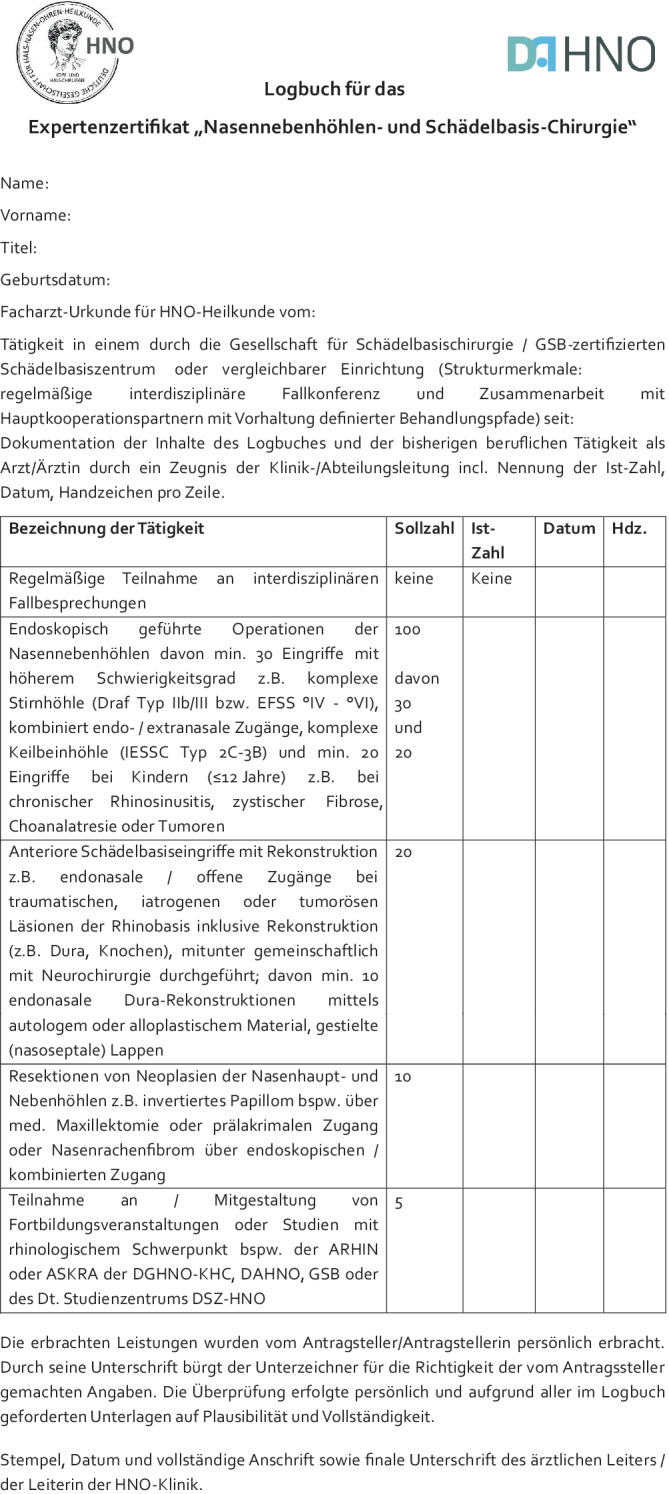
Logbuch mit Kriterien zum Erwerb des Expertenzertifikats „Nasennebenhöhlen- und Schädelbasis-Chirurgie“

### „Nasennebenhöhlen- und Schädelbasis-Chirurgie“

- *Mitgliedschaft* bei der DGHNO-KHC oder DAHNO
- *Facharztqualifikation* für Hals-Nasen-Ohren-Heilkunde (**Urkunde**)
- Mindestens 24 Monate Tätigkeit in einem durch die Gesellschaft für Schädelbasischirurgie/GSB-zertifizierten *Schädelbasiszentrum* oder vergleichbarer Einrichtung (Strukturmerkmale: regelmäßige interdisziplinäre Fallkonferenz und Zusammenarbeit mit Hauptkooperationspartnern mit Vorhaltung definierter Behandlungspfade)
- *Handlungskompetenzen* erbracht in einem Zeitraum von weniger als 60 Monaten/retrospektiv wie folgt (**Logbuch; bei Operationen als Erstoperateur)**:
  - 100 endoskopisch geführte Operationen der Nasennebenhöhlen. Davon min. 30 Eingriffe mit höherem Schwierigkeitsgrad z. B. komplexe Stirnhöhle (Draf Typ IIb/III bzw. EFSS °IV–°VI), kombiniert endo-/extranasale Zugänge, komplexe Keilbeinhöhle (IESSC Typ 2C–3B) und min. 20 Eingriffe bei Kindern (≤ 12 Jahre) z. B. bei chronischer Rhinosinusitis, zystischer Fibrose, Choanalatresie oder Tumoren
  - 20 anteriore Schädelbasiseingriffe mit Rekonstruktion z. B. endonasale/offene Zugänge bei traumatischen, iatrogenen oder tumorösen Läsionen der Rhinobasis inklusive Rekonstruktion (z. B. Dura, Knochen), mitunter gemeinschaftlich mit Neurochirurgie durchgeführt; davon min. 10 endonasale Dura-Rekonstruktionen mittels autologem oder alloplastischem Material, gestielte (nasoseptale) Lappen
  - 10 Resektionen von Neoplasien der Nasenhaupt- und Nebenhöhlen z. B. invertiertes Papillom bspw. über med. Maxillektomie oder prälakrimalen Zugang oder Nasenrachenfibrom über endoskopischen/kombinierten Zugang
  - 5 Teilnahmen an/Mitgestaltungen von Fortbildungsveranstaltungen oder Studien mit rhinologischem Schwerpunkt bspw. der ARHIN oder ASKRA der DGHNO-KHC, DAHNO, GSB oder des Dt. Studienzentrums DSZ-HNO

Die erbrachten Leistungen für die Qualifikation sind durch ein von der ärztlichen HNO-Klinikleitung unterzeichnetes und offiziell gestempeltes Zeugnis entsprechend dem beiliegenden Logbuch nachzuweisen. Durch die Unterschrift der HNO-ärztlichen Klinikleitung bürgt der Unterzeichner für die Richtigkeit der vom Antragssteller gemachten Angaben. Die Überprüfung erfolgte persönlich und aufgrund aller im Logbuch geforderten Unterlagen auf Plausibilität und Vollständigkeit. Die vollständigen Nachweisunterlagen sind vom Antragssteller zu archivieren (z. B. OP-Berichte und Teilnahme-Nachweise) und auf Nachfrage vorzulegen (Abb. 2).

## Antragstellung

Das Antragsformular (Homepage ClarCert) ist mit den geforderten Urkunden und dem Logbuch-Zeugnis digital (pdf-Dokumente) zu senden an:

info@clarcert.com

## Kosten

225 € + Mehrwertsteuer; die Gültigkeit des Zertifikats ist zeitlich nicht begrenzt. Auf Wunsch kann eine Rezertifizierung im Sinne der Aktualisierung erfolgen.
